# Complementary and alternative medicine use among people living with HIV in Shiraz, Southern Iran

**DOI:** 10.3389/fpubh.2023.1206665

**Published:** 2023-10-05

**Authors:** Seyed Hamdollah Mosavat, Mehdi Pasalar, Hassan Joulaei, Vira Ameli, Seyed Taghi Heydari, Ali Mirzazadeh, Mohammad Hashem Hashempur

**Affiliations:** ^1^Research Center for Traditional Medicine and History of Medicine, Department of Persian Medicine, School of Medicine, Shiraz University of Medical Sciences, Shiraz, Iran; ^2^Research Center for Psychiatry and Behavior Science, Shiraz University of Medical Sciences, Shiraz, Iran; ^3^HIV/AIDS Research Center, Institute of Health, Shiraz University of Medical Sciences, Shiraz, Iran; ^4^Centre for Evidence-Based Intervention, Department of Social Policy and Intervention, University of Oxford, Oxford, United Kingdom; ^5^Health Policy Research Center, Shiraz University of Medical Sciences, Shiraz, Iran; ^6^Department of Epidemiology and Biostatistics, University of California, San Francisco, San Francisco, CA, United States

**Keywords:** complementary and alternative medicine, acquired immune deficiency syndrome, HIV, medicinal herbs, Iran, traditional Persian medicine, integrative medicine, quality of life

## Abstract

**Background:**

Living with HIV requires lifelong care to support engagement with and adherence to antiretroviral therapy. The Middle East and North Africa region provides access to ART, but research is lacking on the lived-experiences of people living with HIV. Globally, complementary and alternative medicine (CAM) is increasingly used by patients who need support alongside receiving medical treatment for chronic conditions. This study aims to examine the frequency and reasons behind the use of CAM, as well as identify its associated factors among people living with HIV in Shiraz, Iran.

**Methods:**

In this cross-sectional study, a total of 320 patients (aged 18–70 years) with a confirmed diagnosis of HIV residing in Fars province and diagnosed between 1999 and 2019 were recruited randomly through their clinical record numbers from five HIV treatment centers. They were surveyed on their quality of life and CAM use via the Short-Form Health Survey questionnaire (SF-36) and a semi-structured survey of “CAM use.” The data analysis for this study involved the use of Chi-squared test, independent *t*-test, and multiple logistic regression model.

**Results:**

Of 287 patients, 89.22% reported using CAM in the previous year. CAM use was more prevalent among those with a family history of CAM use (94.3% vs. 81.8%, *p* = 0.023). Frequent reasons for using CAM were reported to be sexual dysfunction (32.4%), depression (28.3%), thirstiness (23.3%), and nausea (17.5%). Quality of life, as measured via the SF-36 questionnaire in all its 8 sub-domains, did not differ among those who used CAM versus those who did not (61.5 ± 27.6 vs. 58.1 ± 30.9, *p* = 0.626).

**Conclusion:**

CAM was used among a majority of people living with HIV in Shiraz, Iran. People who used CAM appeared to experience a similar quality of life relative to those who did not use CAM. Future studies on the modalities of engagement with CAM can improve patient-physician shared decision-making and increase lifelong care options for people living with HIV.

## Introduction

HIV, a significant global health concern, continues to affect millions of individuals worldwide. According to the Joint United Nations Program on HIV/AIDS (UNAIDS), approximately 38 million people live with HIV/AIDS. Sub-Saharan Africa remains the most heavily affected region, accounting for about two-thirds of all people living with HIV globally. The MENA region has a relatively low HIV prevalence compared to other regions. According to UNAIDS, in 2020, an estimated 240,000 people lived with HIV in the MENA region (1–6). Due to factors such as social stigma, limited awareness, and challenges in data collection, HIV/AIDS in the MENA region remains largely hidden and underreported (7). The MENA region also has one of the lowest HIV testing rates globally, contributing to accurate prevalence data gaps. In Iran, it is estimated that around 54,000 people were living with HIV/AIDS in 2020, accounting for approximately 24% of all estimated cases in the MENA region (8). Iran, as one of the countries in this region, faces considerable challenges in managing the spread and impact of HIV on public health (9–13). Alongside conventional medical treatments, there has been growing interest in complementary and alternative medicine (CAM) as a potential avenue for improving the well-being and quality of life of people living with HIV (14–17). Quality of life (QoL) is a multidimensional concept that encompasses various aspects of an individual’s well-being and overall satisfaction with life (18). In the context of HIV patients, QoL refers to the physical, psychological, social, and functional aspects of their daily life experiences. These dimensions are closely interrelated and can significantly impact the overall health and happiness of individuals living with HIV (19).

CAM encompasses a diverse range of therapies and practices that lie outside the realm of conventional medicine (20, 21). Complementary medicine is used alongside conventional treatments to provide additional support and enhance overall health and well-being, while alternative medicine involves the substitution of conventional treatments with non-mainstream approaches. CAM approaches may include herbal medicine, acupuncture, mind–body therapies, dietary supplements, and various other modalities (22–27).

Despite the increasing interest in CAM and its potential benefits for chronic diseases, including HIV, there remains a scarcity of published data on the utilization of CAM among people living with HIV in Iran. The lack of information hinders healthcare professionals and policymakers in understanding the prevalence, types, and factors associated with CAM use within this population. Gaining insights into CAM practices and factors influencing their adoption is crucial for delivering more comprehensive and patient-centered care for individuals living with HIV in Iran (28).

To address this research gap, this study aims to investigate the prevalence and patterns of CAM utilization among individuals living with HIV in Shiraz, a city in Southern Iran. The research will explore various aspects, including demographic characteristics, duration and complications of HIV, and other factors that may be associated with the use of CAM in this specific population. By shedding light on the CAM practices and factors influencing their adoption, this research endeavors to contribute to the existing knowledge base and provide valuable insights for healthcare professionals, policymakers, and researchers involved in HIV care in Iran.

## Materials and methods

In this cross-sectional study, 320 of approximately 2,700 People Living with HIV/AIDS (PLWHA), who were enrolled in care in five medical centers affiliated to Shiraz University of Medical Sciences, in the southern province of Fars, were recruited randomly through their clinical record numbers. Recruitment begun in December of 2019 and terminated in March of 2020. Ethical approval was granted by Shiraz University of Medical Sciences Local Ethics Committee (with the approval code of: IR.SUMS.REC.1398.1399).

The minimum target sample size was calculated as 320 patients, assuming *p* = 60%, one-sided *α* of 0.05, 10% precision of the estimate, and attrition rate of 20%. A patient was included if they confirmed to be living with HIV, had an accessible health profile, resided in Fars province, was aged 18–70 years (*N* = 521), and signed the written informed consent forms. Those who did not consent to participate or were hospitalized were excluded from the study.

Data collection on the use of CAM was through a researcher-developed questionnaire, with its validity and reliability confirmed through expert panel discussions and test–retest methods (Cronbach’s alpha value was 0.83). At first, the general theme and the study’s goals were clarified for patients. The questionnaire consisted of 26 questions and took each patient approximately 10–15 min to complete in a face-to-face interview. Questions included demographic and socioeconomic characteristics, duration since diagnosis with HIV, history of drug use, comorbidities, side-effects, and challenges faced in living with HIV, CD4 level, use of CAM methods before and after HIV diagnosis, family history of CAM use, access to CAM providers, reason for using CAM, frequency of use, modality of use, and beliefs on CAM side-effects and its interaction with antiretroviral medications. In addition, a validated Persian version of Ware and Sherbourne’s “Short-Form Health Survey questionnaire (SF-36)” was used for assessing quality of life (QoL) (29, 30). The SF-36 questionnaire is a self-reported research survey that consists of 36 questions in eight different domains. These questions assess the quality of life in two main subjects of physical and mental health. The score calculation method for SF-36 and its application for health-related quality of life measures in HIV researches previously described in detail (31). In addition, this tool has been used by several researches on HIV patients in Iran (32).

Statistical analysis was carried out using the Statistical Package for Social Sciences (IBM Corp. Released 2019, IBM SPSS statistics for windows, version 26.0. Armonk, NY). Quantitative and qualitative variables were described using mean ± standard deviation (SD) and frequency (percent), respectively. For data visualization, Microsoft^®^ Excel (version 16.43 for MacOS) was used. The Chi-squared test and independent *t*-test (or Wilcoxon rank-sum test) were used to compare qualitative variables between the groups. Additionally, multiple logistic regression model was used to determine the independent predictors for CAM use. Selected variables for inclusion in the regression analysis were based on the bivariate analysis results. If the *p*-value was ≤0.25, the variable was included in the multivariate analysis. A *p*-value of ≤0.05 was considered statistically significant.

## Results

Out of the 320 patients initially interviewed for the study, 33 participants were excluded from the analysis due to missing values in the questionnaires (response rate 89.7%). In total, 287 patients (mean age of 39.12 ± 5.65 years and 55% being male) were included in the final data analysis.

Table 1 shows demographics and characteristics of participants that reported CAM use (89.2%) and no CAM use (10.8%) within the past year. Those who used CAM reported a significantly higher prevalence of family history of CAM than those who did not report CAM use (94.3% vs. 81.8%, *p* = 0.023). The two groups did not statistically differ on all other measured variables (*p* > 0.05). Noticeably, most of the patients in both groups reported equal access to CAM providers, believed that CAM has no side effects, and did not worry about possible interactions between CAM and antiretroviral medications.

**Table 1:** Participant characteristics: reporting CAM use vs. no CAM use within the past year.

Parameter	CAM users (*n* = 265)	non-CAM users (*n* = 22)	*p*-value
Age	40.8 ± 8.1	42.0 ± 9.7	0.577
Gender^¥^MaleFemale	143 (54.6)122 (45.4)	15 (68.2)7 (31.8)	0.198
Time since diagnosis duration (year)	6.6 ± 4.1	5.6 ± 4.1	0.32
Residency^¥^RuralUrban	30 (11.3)235 (88.7)	5 (22.7)17 (77.3)	0.102
Education level^¥^IlliteratePrimary schoolSecondary schoolUniversity	8 (3)182 (68.7)63 (23.8)12 (4.5)	015 (68.2)7 (31.8)0	0.532
CD4 ≥ 350 (measured in 6 months prior to the study)^¥^	169 (65.5)	13 (61.9)	0.739
History of CAM use in family^¥^	250 (94.3)	18 (81.8)	0.023^*^
Access to CAM providers^¥^	247 (93.2)	22 (100)	0.207
Opinion about CAM side effects^¥^NeverRarelySometimesOftenAlwaysNo idea	174 (65.7)21 (7.9)24 (9.1)5 (1.9)5 (1.9)36 (3.6)	13 (59.1)4 (18.2)1 (4.5)004 (18.2)	0.521
CAM and anti-viral drugs interactions^¥^Synergistic effectNeutralizing effectInducing side effectNo idea	12 (4.5)76 (28.7)41 (15.5)136 (51.3)	1 (4.5)3 (13.6)4 (18.2)14 (63.7)	0.502

Mean ± Standard Deviation analyzed by independent *t*-test.

^¥^ Frequency (Percent) analyzed by Chi-square test.

^*^ Statistically significant.

Table 2 shows the frequency of CAM use and the frequently reported reasons for choosing CAM to address a variety of psychosocial or health needs. The only CAM method reported by the participants was the use of herbal medicines, which consisted of: Chamomile (24.15%), Borage (15.47%), Chicory (9.43%), Thyme (8.67%), Mint (7.2%), and other herbs (18.87%). The most frequently reported psychosocial and medical problem experienced by participants was depression (62.7%), fatigue (57.5%), muscle pain (55.4%), and joint pain (50.5%). CAM was reported to be used for sexual dysfunction (32.4%), depression (28.3%), thirst (23.3%), and nausea (17.5%), respectively.

**Table 2:** Frequency of CAM use and the frequently reported reasons for choosing CAM.

Complaint	Frequency (%)^‡^	CAM use [*n* (%)]^†^	Complaint	Frequency (%)^‡^	CAM use [*n* (%)]^†^
Depression	180 (62.7)	51 (28.3)	Cough/Dyspnea	94 (32.8)	8 (8.5)
Fatigue	165 (57.5)	15 (9.1)	Thirst	86 (30)	20 (23.3)
Muscle pain	159 (55.4)	9 (5.7)	Dizziness	84 (29.3)	4 (4.8)
Joint pain	145 (50.5)	7 (4.8)	Night sweats	78 (27.2)	8 (8.5)
Headache	131 (45.6)	9 (6.9)	Nausea	63 (22)	11 (17.5)
Cognitive impairment	130 (45.3)	4 (3.1)	Fever/Chills	61 (21.3)	2 (3.3)
Bloating	120 (41.8)	3 (2.5)	Constipation	59 (20.6)	4 (6.8)
Numbness	119 (41.5)	2 (1.7)	Abdominal pain	54 (18.8)	3 (5.6)
Itchiness	109 (38)	0	Anxiety	51 (17.8)	0
Sexual dysfunction	108 (37.6)	35 (32.4)	Anorexia	35 (12.2)	0
Insomnia	96 (33.4)	1 (1)	Other	115 (40.1)	7 (6.1)

^‡^ Out of 278 patients.

^†^ Out of patients with a complaint.

The two groups did not statistically differ on quality of life sub-domains within SF-36 QoL questionnaire (*p* > 0.05) (see Table 3).

**Table 3:** Comparison of QoL in CAM users vs. non-CAM users.

Variable	CAM users (*n* = 265)^§^	non-CAM users (*n* = 22)^§^	*p* ^*^
Physical health (overall)	61.5 ± 27.6	58.1 ± 30.9	0.626
Physical functioning	66.3 ± 33.5	63.0 ± 35.5	0.673
Role limitation due to physical problems	63.6 ± 45.4	61.4 ± 45.5	0.823
Bodily pain	69.3 ± 35.9	64.9 ± 38.6	0.609
General health	46.8 ± 23.3	43.2 ± 22.9	0.479
Correlated physical health (PCSc)	52.16 ± 9.37	51.12 ± 10.12	0.629
Mental health (overall)	58.4 ± 29.6	56.6 ± 34.0	0.81
Role limitation due to emotional problems	50.9 ± 47.9	62.1 ± 48.6	0.309
Vitality	56.5 ± 29.8	50.9 ± 34.9	0.474
Emotional wellness	56.5 ± 27.3	49.7 ± 30.8	0.327
Social functioning	69.7 ± 32.1	63.6 ± 36.2	0.452
Correlated mental health (MCSc)	40.57 ± 14.65	38.33 ± 16.02	0.504

^§^ Mean ± standard deviation; ^*^ Independent *t*-test.

For controlling potential confounders, a regression analysis model was used. Patients who were resident in rural area (*p* = 0.045, Odds ratio = 3.218, and 95% confidence intervals for odds 1.027–10.082) and had history of CAM use in their family (*p* = 0.018, Odds ratio = 4.829, and 95% confidence intervals for odds 1.315–17.741) were significantly more likely to use CAM. However, MCSc and PCSc (as the most important items of QoL variables), and patients’ sex were not independent predictors of CAM use (*p* = 0.504, 0.526, and 0.150; respectively).

## Discussion

Our data showed that 89.2% of people living with HIV in Southern Iran reported CAM use, which is higher than the commonly reported average of 60% CAM use for managing HIV-related psychosocial and health concerns (33). Among different examined variables, residency in rural areas and history of CAM use in patients’ family were independent predictors for CAM use. Understanding the prevalence and types of CAM therapies utilized by people living with HIV in Shiraz, as well as the factors influencing their decision to use CAM, can inform healthcare providers about the needs and preferences of patients, foster open communication, and support the development of integrated and holistic approaches to HIV management. Ultimately, the findings from this study can contribute to optimizing patient care and promoting the well-being of individuals living with HIV in Southern Iran.

Recent evidence from Thailand showed that the increasing and widespread trend of CAM use can be partially explained by easier and cheaper access and adaptation to indigenous culture or folklore in many countries, in addition to population aging with growing chronic and debilitating diseases, medication burden and side effects, and an increase in access to online health information (34). Previous studies from South Africa and the US suggested that some HIV patients resort to CAM and traditional medicine when they experience side effects from the antiretrovirals (35). Importantly, however, the reported rates of CAM use are affected by the different CAM methods chosen across countries and regions (35). Given the variety of CAM approaches available in Iran as part of traditional Persian medicine, with some administered through lesions (36), it is indeed significant that the only method chosen by people living with HIV in the Fars province is herbal medicines, which do not possess any risk of HIV transmission during the administration.

CAM can be a useful approach for people living with HIV to improve wellbeing, stress, anxiety, fear, depression, pain, fatigue, fever, and neuropathy (35, 37–39). However, antiretroviral guidelines suggest patients disclose CAM use due to the possibility of adverse interactions between medications. Yet, studies show that most patients do not acknowledge the use of CAM in managing their treatment for HIV/AIDS (35, 40), HIV-related psychosocial challenges and ART side-effects (33). Evidence from clinical settings in the US shows that the most frequent problems for which CAM is used are dermatological conditions (45%), nausea (44%), depression (36%), insomnia (36%), and weakness (34%) (41). In-line with this body of evidence, we observed depression, muscle pain, joint pain, and fatigue in more than 50% of patients. The most commonly reported concern in this study was depression (62.7%), which was also reported to be the second major reason for using CAM. The other major reasons were sexual dysfunction, thirstiness, and nausea. Importantly, psychosocial and chronic somatic concerns of people living with HIV may interact. For example, depression and loss of appetite have been previously associated with unresponsive pain (42, 43). In addition, sexual dysfunction is a well-known symptom of depression, causing a complex interplay with psychological states and experience of pain (44).

Systematic review evidence shows that CAM use is associated with female sex, ethnicity, education level, economic status, the severity of symptoms, higher viral load, and longer duration of HIV infection (33). Additionally, health insurance coverage, negative attitude of health providers toward CAM use, negative attitude of patients toward ART use, and lower CD4 cell count can impact the rate and tendency to turn to CAM, showing a large heterogeneity of determinants (45–48). In our study, there was no difference between the two groups in the majority of the characteristics examined, including gender, socioeconomic status, demographics and other characteristics. Although, residency in rural areas was an independent predictor for CAM use. Previous studies on people living with HIV found no significant differences in CAM use by urban vs. rural residents or did not assess this variable (49–51). Given the free provision of ART in Iran, health insurance and financial status do not determine the accessibility of ART and the choice of CAM cannot be due to unaffordability of ART. As opposed to financial, the challenges of living with HIV in Iran are largely socioecological and driven by stigma and low societal awareness of HIV (52). As such, socioecological determinants could be investigated in the future. The only significant factor determining the tendency to use CAM was previous history of CAM use within family. This effect on the tendency to use CAM has been previously reported for other chronic diseases in other settings (53). For example, in a study of parents and caregivers of HIV children in Nigeria, 37.1% of the interviewees declared that relatives, friends, and neighbors’ opinion influenced their willingness and openness toward CAM (54). As such, the pattern of CAM use appears to be different among people living with HIV according to their distinct set of cultural beliefs, myths, evidence, and knowledge about CAM.

While it is reported that CAM use has been significantly associated with higher ART adherence in developed counties (48), the high prevalence of CAM use may interfere with adherence to concomitant use of ART (55), due to overestimating the therapeutic benefits over palliative ones, or underestimating the harms of CAM and economic issues, as people living with HIV are given limited information on the safety of CAM (56). Similarly, in our study we observed that people living with HIV are not concerned about any side effects of CAM or possible interactions with ART. It is important to note that herbal medicine is the only modality of CAM that was reported by our participants, and as such the concern for side effects could indeed be minimal. Nevertheless, there is a need for more studies to investigate the benefits, side effects and toxicity of various modalities of CAM for people living with HIV (16, 57–59), inform intervention programs and supporting decisions by both physicians and patients. In addition, understanding the varied perspectives of people living with HIV in different cultural and social contexts could further inform unbiased choices that support and strengthen patient-physician relationship in ways that CAM use is understood and adherence to ART is simultaneously increased (45, 60–63).

Globally, patients with lifelong chronic diseases such as cancer and HIV/AIDS use CAM to improve their quality of life and reduce the challenges faced psychosocially and medically. Improving wellbeing, general physical condition, preventing or improving symptoms, and alleviating side-effects of antiretroviral medications, is expected to improve the quality of life of people living with HIV, rather than directly treating HIV (35, 62). However, in our study, we observed no difference with regards to quality of life between those who used CAM and those who did not. Similarly, in a study from the United States, CAM use did not drive improvements with regards to physical functioning, general health, overall health, overall quality of life, and social support (49). Any impact of CAM on reducing depressive symptoms should be further investigated, especially considering the various modalities and cultural contexts of CAM practice (33). While it appears that using CAM is not an independent predictor of quality of life, as measured quantitatively, it may be illuminating to investigate the impact of CAM through in-depth qualitative and theory-driven studies, in which benefits through reasoning and reflections of participants can be further explored. Larger quantitative studies can also be useful for multivariate models that can adjust for any confounding factors and perform sub-group analysis.

### Study limitations

Despite the valuable insights gained from this study, there are several limitations that should be acknowledged. Firstly, the study sample size was relatively small, which may limit the generalizability of the findings to the larger population of people living with HIV in Southern Iran. The small sample size of this study did not allow sub-group analysis. Future studies with larger sample sizes are needed to confirm and validate the results.

Secondly, the study was conducted in a specific geographic region, Shiraz, Southern Iran, which may limit the generalizability of the findings to other regions within Iran or other countries. Different cultural, social, and healthcare system factors could influence the patterns of CAM use among people living with HIV in different contexts. Therefore, caution should be exercised when extrapolating the findings to other populations.

Another limitation is the use of self-reported data, which is subject to recall bias and social desirability bias. Participants may have underreported or overreported their CAM use due to various reasons, including stigma associated with CAM use or the desire to please the researchers. Future studies could consider employing objective measures, such as biomarker analysis, to validate the self-reported CAM use.

Furthermore, this study focused only on people living with HIV who were receiving ART. This excludes individuals who may have chosen CAM over ART or those who are not accessing medical care. Therefore, the findings may not fully capture the perspectives and experiences of all people living with HIV in the region. Other limitation of this study is that dietary ingredients and spices used for culinary purposes were not considered as a type of CAM use. It may have excluded important information about the role of diet and nutrition in managing HIV.

### Recommendations

To anticipate or minimize these limitations in future research, a larger and more diverse sample should be recruited to enhance the generalizability of the findings. Employing a multi-center approach that includes participants from different regions within Iran would provide a more comprehensive understanding of CAM use among people living with HIV in the country. Additionally, combining quantitative and qualitative research methods can provide a more nuanced understanding of the factors influencing CAM use and its impact on the lives of individuals with HIV. Future studies could consider incorporating dietary information to provide a more comprehensive understanding of CAM use among HIV patients. Finally, using objective measures to assess CAM use and exploring the perspectives of individuals who are not accessing conventional medical care would provide a more comprehensive picture of CAM utilization in this population.

## Conclusion

We found a significantly higher rate of CAM use among people living with HIV in Iran than previously reported in other settings. Most of the socioeconomic or demographic differences did not appear to determine the tendency to use CAM. The only factors independently correlated with CAM use was a family history of CAM use and residency in rural area. A variety of other reasons, such as socioecological influences, that were not measured could be plausibly contributing to the willingness to choose CAM. Qualitative and theory-based research designs could investigate patient beliefs, behaviors, and socioecological factors that impact relation to CAM among people living with HIV. Moreover, research on toxicity levels, interactions with prescribed drugs, and therapeutic effects of CAM are needed to help improve decision-making for both patients and physicians.

## Data availability statement

The raw data supporting the conclusions of this article will be made available by the corresponding authors, without undue reservation.

## Ethics statement

This study was reviewed and approved by Shiraz University of Medical Sciences Local Ethics Committee (with the approval code of: IR.SUMS.REC.1398.1399). The patients/participants provided their written informed consent to participate in this study.

## Author contributions

SM, MP, MHH, SH, and HJ: conception and designing of study. SM and MHH: data collection and supervision. SH and MP: data analysis. MP, VA, and AM: manuscript drafting. MP, MHH, VA, AM, and HJ: critical revision. All authors read and approved the final version of the manuscript.
